# Long noncoding RNA papillary thyroid carcinoma susceptibility candidate 3 (PTCSC3) inhibits proliferation and invasion of glioma cells by suppressing the Wnt/β-catenin signaling pathway

**DOI:** 10.1186/s12883-017-0813-6

**Published:** 2017-02-10

**Authors:** Shujun Xia, Ri Ji, Weiwei Zhan

**Affiliations:** Ultrasound Department, Rui Jin Hospital Shanghai Jiao Tong University School of Medicine, 197 Rui Jin Er Road, Huang Pu District, Shanghai, Zip code: 200025 People’s Republic of China

**Keywords:** LncRNA PTCSC3, Glioma, Proliferation, Invasion, EMT, Wnt signal

## Abstract

**Background:**

The dysregulation of long noncoding RNAs (lncRNAs) has been identified in a variety of cancers. An increasing number of studies have found the critical role of lncRNAs in the regulation of cellular processes, such as proliferation, invasion and differentiation. Long noncoding RNA papillary thyroid carcinoma susceptibility candidate 3 (PTCSC3) is a novel lncRNA that was primarily detected in papillary thyroid carcinoma. However, the biological function and molecular mechanism of lncRNA PTCSC3 in glioma are still unknown.

**Methods:**

The expression level of lncRNA PTCSC3 in human microglia and glioma cell lines was examined using quantitative real-time polymerase chain reaction (qRT-PCR). The influence of lncRNA PTCSC3 on cell proliferation were studied using the cell counting kit-8, and cell cycle and apoptosis were analyzed by flow cytometry assays. The migration and invasion abilities were investigated by transwell and wound healing assays. The target genes of lncRNA PTCSC3 were explored by qRT-PCR, immunofluorescence and western blot.

**Results:**

LncRNA PTCSC3 was significantly downregulated in glioma cell lines. The overexpression of lncRNA PTCSC3 suppressed proliferation and induced apoptosis in U87 and U251 cells. Additionally, the overexpression of lncRNA PTCSC3 inhibited the migration and invasion of U87 and U251 cells. Moreover, lncRNA PTCSC3 inhibited the epithelial-mesenchymal transition of U87 cells. The study also demonstrated that LRP6, as a receptor of the Wnt/β-catenin pathway, was a target of lncRNA PTCSC3. By evaluating the expression levels of Axin1, active β-catenin, c-myc, and cyclin D1, the study indicated that lncRNA PTCSC3 inhibited the activation of the Wnt/β-cateninpathway through targeting LRP6.

**Conclusions:**

LncRNA PTCSC3 inhibits the proliferation and migration of glioma cells and suppresses Wnt/β-catenin signaling pathway by targeting LRP6. LncRNA PTCSC3 is a potential therapeutic target for treatment of glioma.

**Electronic supplementary material:**

The online version of this article (doi:10.1186/s12883-017-0813-6) contains supplementary material, which is available to authorized users.

## Background

Glioma appears to be one of the most common types of primary brain tumors in adults [1, 2]. Characterized by rapid progression, patients with glioma are most likely diagnosed at advanced stages, and the prognosis remains poor [3], posing a large threat to human health. Although some advances in comprehensive treatment as well as early diagnosis have been made, only a few patients have experienced the expected effects when translated to the clinic [4, 5]. Thus, it is imperative to explore the mechanisms concerning glioma formation and progression and to establish diagnostic and therapeutic targets for optimized management of glioma.

Long noncoding RNAs (lncRNAs) are defined as non-protein coding transcripts longer than 200 nucleotides. Recently, lncRNAs have gained much attention in the field of molecular biology. Increasing evidence indicates that lncRNAs are involved in diverse biological processes, including cell proliferation, differentiation, apoptosis, development and immune responses [6, 7]. Papillary thyroid carcinoma susceptibility candidate 3 (PTCSC3) is an intergenic long noncoding RNA gene (lincRNA) located at 14q.13.3, which was newly identified as thyroid specific [8]. Subsequently, lncRNA PTCSC3 was reported to be a tumor suppressor in thyroid cancer [9], and the mechanism study demonstrated that lncRNA PTCSC3 reduced cell motility and invasiveness by downregulating the S100A4 pathway [10]. However, little is known about the role of lncRNA PTCSC3 in other malignancies.

Our study was performed to assess the expression of lncRNA PTCSC3 in glioma cells and to evaluate its role and mechanism in tumor cell proliferation, invasion and migration. This is the first time that lncRNA PTCSC3 has been assessed in glioma. We assessed the expression level of lncRNA PTCSC3 in human microglia and glioma cell lines. Additionally, we demonstrated that lncRNA PTCSC3 overexpression suppressed proliferation, migration and invasion and inhibited the epithelial-mesenchymal transition (EMT) by suppressing the Wnt/β-catenin signaling pathway in glioma.

## Methods

### Cell culture

Several glioma cell lines (U87, U251, SHG44 & SHG139) were purchased from the Cell Bank Type Culture Collection of the Chinese Academy of Sciences (Shanghai, China). Human microglia was purchased from the Scinencell Research Laboratories (Carlsbad, CA, USA). Human astrocyte was purchased from Lonza (Basel, Switzerland) and cultured in AGM™ Astrocyte Growth Medium. Other cells were cultured in DMEM (Gibco, Carlsbad, CA, USA) supplemented with 10% of fetal bovine serum (Gibco) and 1% of penicillin-streptomycin at 37 °C, 5% CO_2_ humidified atmosphere.

### RNA extraction and quantitative reverse transcriptase-polymerase chain reaction (qRT-PCR)

Total RNA was extracted from cell lines with TRIzol reagent (Invitrogen, Carlsbad, CA, USA) and the concentration was measured by nanodrop spectrophotometer. One microgram of total RNA was reversely transcribed into first-strand cDNA using a Reverse Transcription Kit (Takara, Dalian, China). PCR was performed in VIIA7 system (Applied Biosystems, California, USA) with SYBR® Premix Ex Taq^TM^ II Kit (Takara, Dalian, China). GAPDH was used as an internal control. Gene primers are listed in Table 1. Comparison Ct (2-△△Ct) method was used to analyze the data.

**Table 1:** Sequence of primers used for qRT-PCR

Gene		Sequence (5′–3′)
lncRNA PTCSC3	Forward Primer	GGCTTGAACAATCTTCCCACCTT
	Reverse Primer	TTTGGCAACACCCTCACAGACAC
MMP1	Forward Primer	AAAATTACACGCCAGATTTGCC
	Reverse Primer	GGTGTGACATTACTCCAGAGTTG
MMP2	Forward Primer	CCCACTGCGGTTTTCTCGAAT
	Reverse Primer	CAAAGGGGTATCCATCGCCAT
MMP9	Forward Primer	AGACCTGGGCAGATTCCAAAC
	Reverse Primer	CGGCAAGTCTTCCGAGTAGT
MMP13	Forward Primer	ACTGAGAGGCTCCGAGAAATG
	Reverse Primer	GAACCCCGCATCTTGGCTT
E-cadherin	Forward Primer	CGAGAGCTACACGTTCACGG
	Reverse Primer	GGGTGTCGAGGGAAAAATAGG
Fibronectin	Forward Primer	CGGTGGCTGTCAGTCAAAG
	Reverse Primer	AAACCTCGGCTTCCTCCATAA
Snail	Forward Primer	TCGGAAGCCTAACTACAGCGA
	Reverse Primer	AGATGAGCATTGGCAGCGAG
ZEB1	Forward Primer	GATGATGAATGCGAGTCAGATGC
	Reverse Primer	ACAGCAGTGTCTTGTTGTTGT
FZD8	Forward Primer	ATCGGCTACAACTACACCTACA
	Reverse Primer	GTACATGCTGCACAGGAAGAA
LRP6	Forward Primer	TTTATGCAAACAGACGGGACTT
	Reverse Primer	GCCTCCAACTACAATCGTAGC
Axin1	Forward Primer	GACCTGGGGTATGAGCCTGA
	Reverse Primer	GGCTTATCCCATCTTGGTCATC
C-myc	Forward Primer	GGCTCCTGGCAAAAGGTCA
	Reverse Primer	CTGCGTAGTTGTGCTGATGT
Cyclin D1	Forward Primer	TGGAGCCCGTGAAAAAGAGC
	Reverse Primer	TCTCCTTCATCTTAGAGGCCAC
GAPDH	Forward Primer	AAGGTGAAGGTCGGAGTCAAC
	Reverse Primer	GGGGTCATTGATGGCAACAATA

### Lentivirus transfection

The lncRNA PTCSC3 was ligated into pLVX-IRES-puro to construct the lncRNA PTCSC3 overexpression plasmid. The pRUF-IRES-puro-PTCSC3 and pLVX-IRES-puro constructs were transfected into the HEK293T viral packaging cell line together with psPAX2 and pMD2.G plasmids. Forty-eight hours after transfection, the supernatant was collected and used to infect the cells.

### CCK-8 assay

U87 and U251 were selected as the appropriate cell lines for gain-of-function experiments. The two cell lines were seeded into 96-well plates at a density of 1 × 10^4^ cells/well 48 h after transfection and cultured at 37 °C, 5% CO_2_ incubator. The viabilities of cells were assessed with the cell counting kit-8 (CCK-8; Dojindo Molecular Technologies, Japan) at four time points (12, 24, 48 & 72 h). The absorbance was detected by a TECAN infinite M200 plate reader at 450 nm.

### Colony formation assay

For colony formation assay, 500 cells were seeded in each well of a six-well plate 48 h after transfection and cultured at 37 °C, 5% CO2 incubator with the medium being changed every 2 days. On the 7th day, the cultural media was removed, and cells were washed twice with PBS. After that, colonies were fixed in methanol for 20 min and stained with 1% crystal violet (Sigma) for 30 min, and then washed. Colonies were counted and photographed.

### Flow cytometry assay

Fourty-eight hours after transfection, U87 and U251 cells were collected for cell cycle and apoptosis analysis. The cell cycle analysis was performed using the Cell Cycle Analysis Kit (Beyotime biotechnology, Jiangsu, China) according to the manufacturer’s instructions. The apoptosis assay was performed using the Alexa Fluor® 488 Annexin V/Dead Cell Apoptosis Kit (Thermo Fisher Scientific, Waltham, MA, USA). The cells were detached by trypsinase, centrifuged at 1000 g for 5 min and then resuspended in 100 μL of binding buffer with 5 μL of Annexin v. After that, 1 μL of propidium iodide was added and mixed, followed by 15 min’ incubation in dark. Subsequently, 400 μL of binding buffer was added to resuspend the cells, which were analyzed by Gallios Flow Cytometry (Beckman Coulter, USA). The experiments were repeated triple times.

### Transwell assay

Cell invasion abilities were performed with transwell assay using transwell chambers with 8-μm pores (Costar, Corning, NY, USA). In invasion assay, the upper side of the insert membrane was coated with matrigel (BD Biosciences, New Jersey, USA). A number of 1 × 10^4^ cells in 200 μl of serum-free medium were added into the upper chamber and 600 μl of medium with 5% FBS as chemoattractant was added into the lower chamber. After 24 h’ incubation, the cells on the upper surface of the insert membrane were removed using cotton swab. The cells on the lower surface of the insert membrane were fixed in methanol for 20 min and then stained with 1% crystal violet for 30 min. The number of cells on the lower surface of the membrane was calculated with a microscope and then photographed. All experiments were performed in triplicate.

### Scratch assay

Cells were seeded in each well of 6-well plates and cultured until 100% confluence. A scratch was performed using pipette tips and then serum-free medium was replaced. The scratch closing procedure was observed in 24 h and images were taken after 24 h’ incubation.

### Western blot analysis

Total protein was extracted from collected cells with RIPA buffer containing phenylmethanesulfonylfl uoride (PMSF). After a 30-min reaction on ice, the protein lysate was centrifuged at 13000 rpm for 15 min and the supernatant was collected. The protein concentration was measured by the BCA Protein Assay Kit (Pierce, Illinois, USA). Ten microgram of each protein was subjected to 8% SDS-PAGE and transferred to PVDF membranes, which was then blocked with 5% BSA in TBST (TBS containing 0.1% Tween-20) at room temperature for 1 h and incubated with the corresponding primary antibodies diluted in blocking buffer at 4 °C overnight. After washing with PBST (PBS containing 0.1% Tween), secondary antibodies was added for incubation at room temperature for 1 h. The western blot images were captured using the LI-COR Odyssey Scanner. Primary antibodies recognizing human E-cadherin, Fibronectin, LRP6, Axin1, active catenin, C-myc, Cyclin D1 (mouse mAb, 1:1000, Abcam, Cambridge, MA, USA) and GAPDH (rabbit mAb, 1:2000, Cell Signaling Technology, Danvers, MA, USA) were used.

### Immunofluorescence assay

U87 cells were seeded in 35-mm cell culture dishes (NEST Biotech, Wuxi, China). The cells were fixed with 4% PFA in PBS for 20 min and permeabilized with 0.5% Triton X-100 for 5 min at room temperature. After washing in PBS, cells were blocked with 5% BSA for 1 h and incubated with primary antibodies against E-Cadherin and Fibronectin (1:200, Abcam, Cambridge, MA, USA) overnight at 4 °C, and then detected using Alexa fluor 546-conjugated anti-mouse IgG secondary antibodies, followed by a 2-mg/mL solution of 4′, 6-diamidino-2-phenylindole (DAPI; Sigma–Aldrich, St. Louis, MO, USA) in PBS for nuclear staining. Cells were visualized under a confocal microscope (Leica, Solms, Germany).

### RNAi

For RNAi-mediated knockdown experiment, lncRNA PTCSC3-specific siRNA and scramble negative control siRNA (ScrsiRNA) were purchased from GenePharma Co., Ltd (Shanghai, China). The sequences were as follow: lncRNA PTCSC3-siRNA, sense, 5′-AUCUUUUGCAUUAAUCUCCCU-3′, antisense, 5′-GGAGAUUAAUGCAAAAGAUGG-3′; ScrsiRNA, sense, 5′-UUCUCCGAACGUGUCACGUTT-3′, antisense, and 5′-ACGUGACACGUUCGGAGAATT-3′. U87 cells were transfected with 100 nM lncRNA PTCSC3 specific siRNA or scrsiRNA using Lipofectamine^TM^2000 (Thermo Fisher Scientific, Waltham, MA, USA) according to the manufacturer’s protocol. The transfection efficiency was confirmed by qRT-PCR ﻿(﻿See Additional file 1: Figure S1).

### Statistical analysis and quantification

All statistical analyses were performed using Statistical Program for Social Sciences 19.0 software (SPSS, Chicago, IL, USA) and GraphPad Prism 5.0 (GraphPad Software, LaJolla, CA, USA). Data were presented as the means ± SD. The significance of differences between groups was estimated by Student’s *t*-test (two-sided, unpaired). *P* < 0.05 was considered to indicate a statistically significant difference. All experiments were repeated at least three times.

## Results

### LncRNA PTCSC3 overexpression inhibits the proliferation and induces apoptosis in glioma cells

**Fig. 1:**
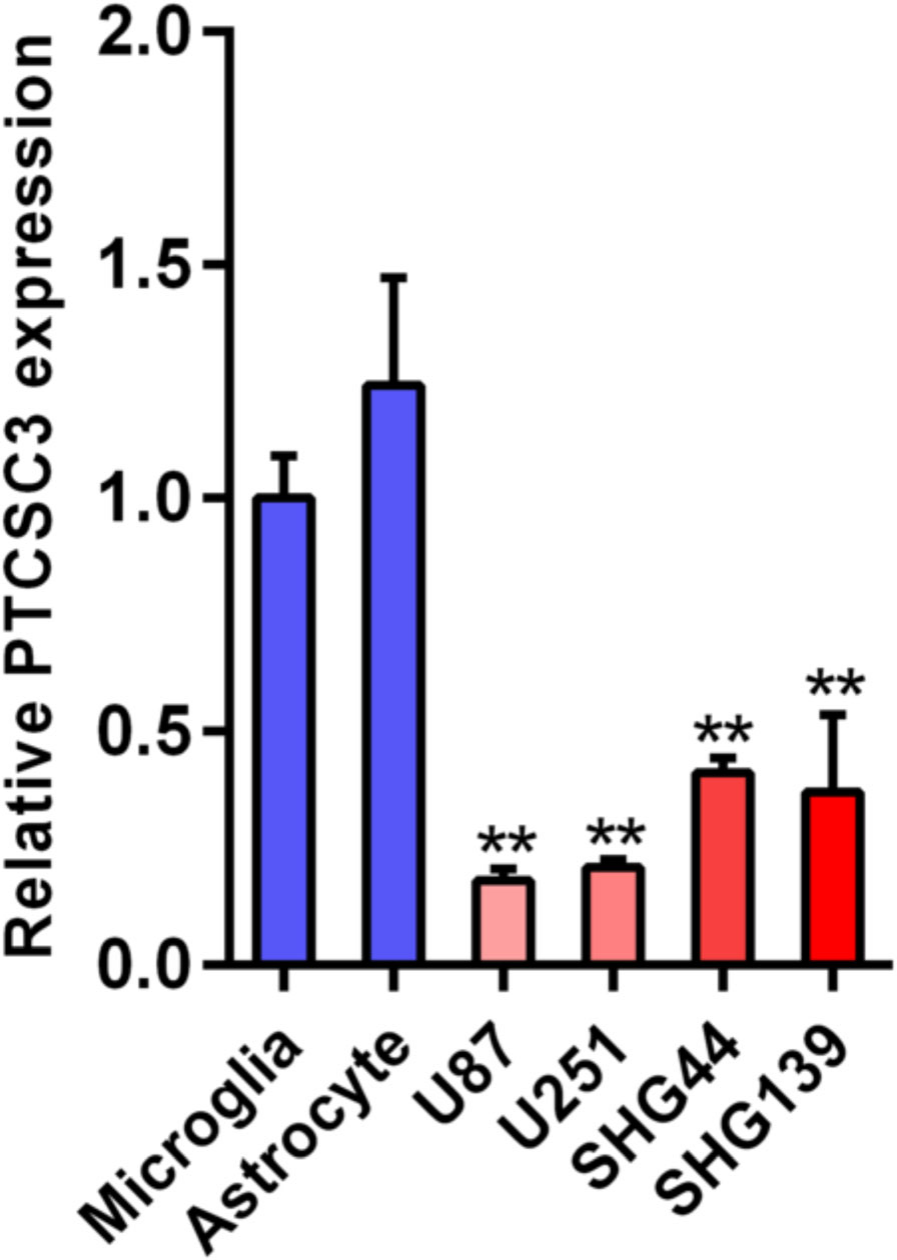
Expression of lncRNA PTCSC3 in human microglia and glioma cell lines. qRT-PCR analysis of the expression levels of lncRNA PTCSC3 in human microglia, astrocyte and glioma cell lines (U87, U251, SHG44 & SHG139). The values were normalized to GAPDH mRNA expression. Data are expressed as the means ± S.D of three independent experiments. “**” indicates *P* < 0.01

**Fig. 2:**
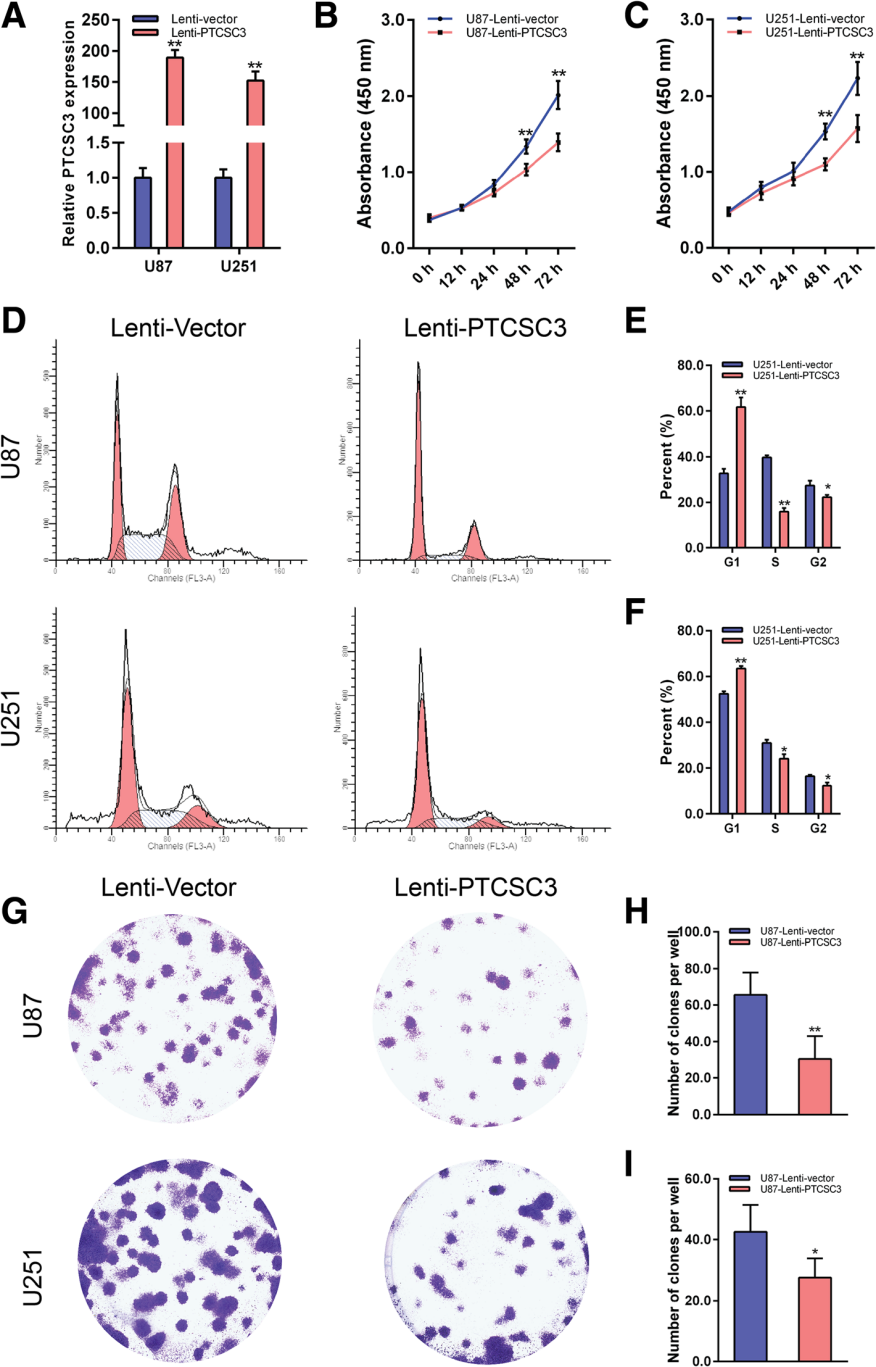
LncRNA PTCSC3 overexpression inhibits the proliferation of U87 and U251. **a** qRT-PCR analysis of lncRNA PTCSC3 expression levels in U87 and U251 infected with Lenti-Vector or Lenti-PTCSC3. CCK-8 assays were performed to determine U87 (**b**) and U251 (**c**) cell proliferation after 12 h, 24 h, 48 h and 72 h infected with Lenti-Vector or Lenti-PTCSC3. **d** Representative flow cytometry images of the cell cycle in U87 and U251 cells infected with Lenti-Vector or Lenti-PTCSC3. The cell cycle results quantified in of U87 (**e**) and U251 (**f**) cells are presented as the percentage of total cells. **g** Colony formation assay was performed to determine the proliferation of U87 and U251 cells infected with Lenti-Vector or Lenti-PTCSC3. The colonies were captured and counted. Colony formation assay results of U87 (**h**) and U251 (**i**) cells are presented as histograms. Data are expressed as the means ± S.D of three independent experiments. “*” indicates *P* < 0.05, “**” indicates *P* < 0.01

**Fig. 3:**
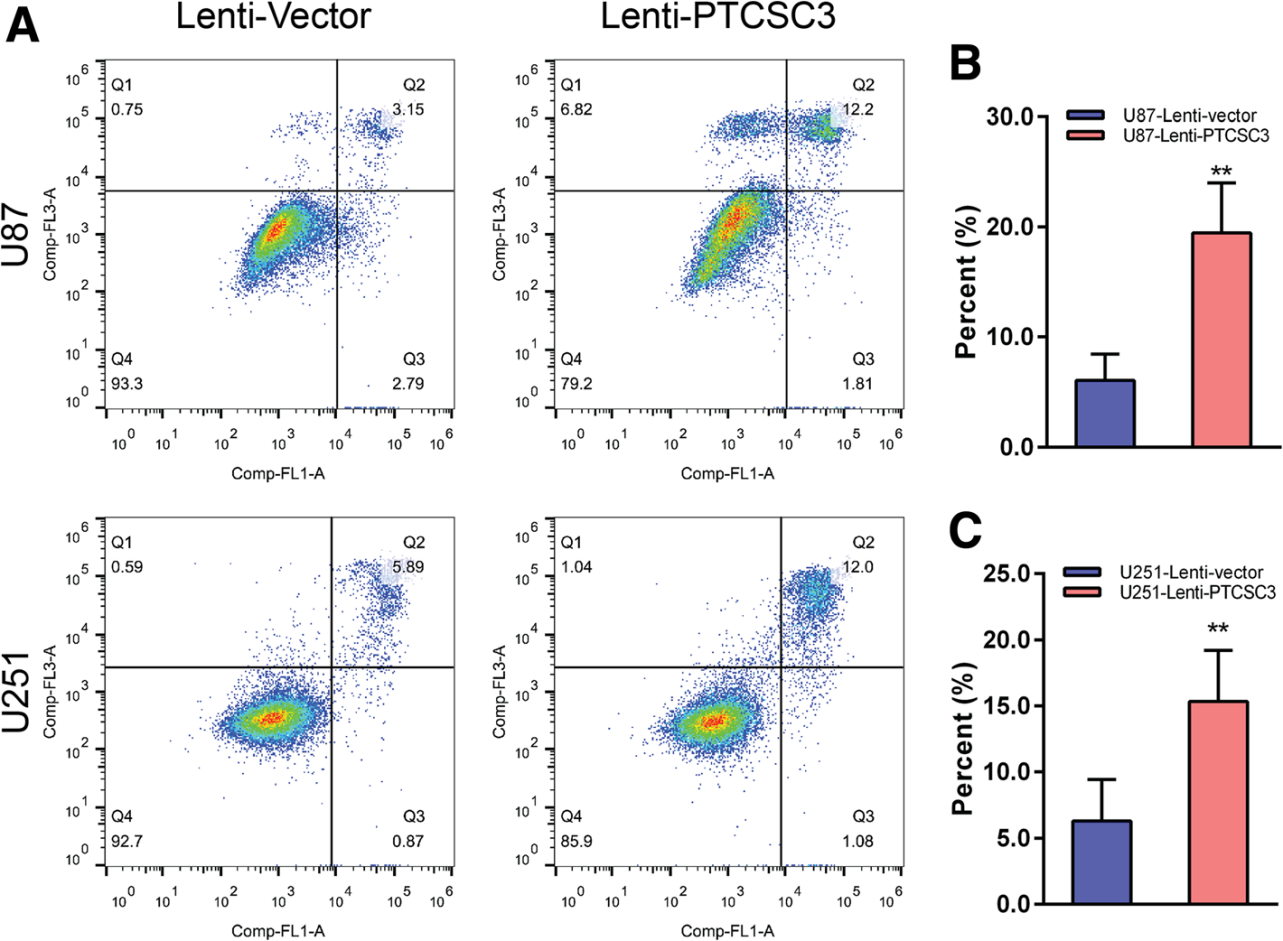
LncRNA PTCSC3 overexpression induces apoptosis in U87 and U251. **a** Representative flow cytometry images of apoptosis of U87 and U251 cells infected with Lenti-Vector or Lenti-PTCSC3. The percentage of apoptotic U87 (**b**) and U251 (**c**) cells is presented as histograms. Data are expressed as the means ± S.D of three independent experiments. “*” indicates *P* < 0.05, “**” indicates *P* < 0.01

**Fig. 4:**
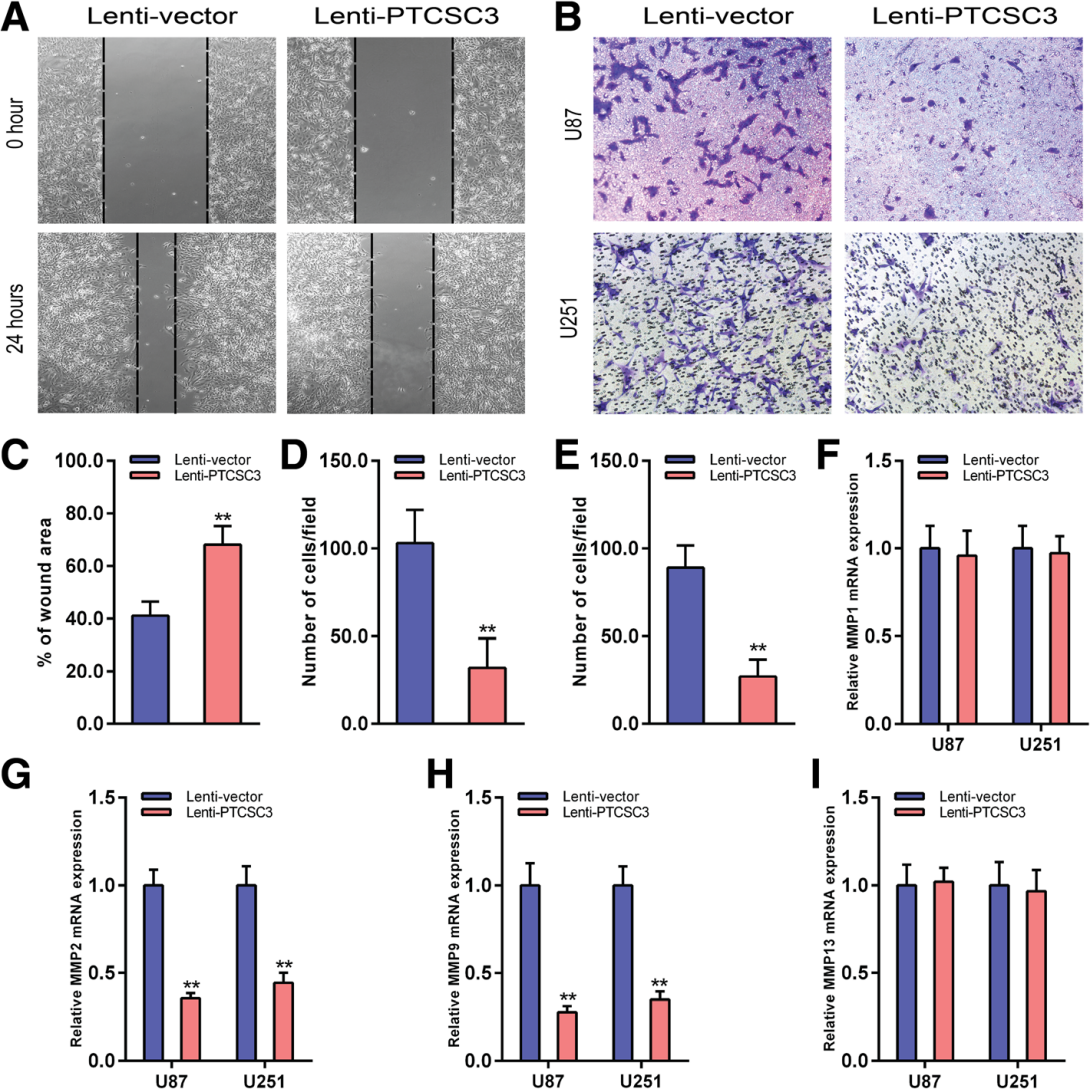
LncRNA PTCSC3 overexpression inhibits the migration and invasion of U87 cells. **a** Wound healing assay was used to determine the migration ability of U87 cells with or without lncRNA PTCSC3 overexpression. Representative images of 0 h and 24 h of three repeated experiments are presented. **b** Transwell assay was performed to determine the invasion ability of U87 and U251 cells with or without lncRNA PTCSC3 overexpression. The representative images of invasive cells in the lower chamber stained with crystal violet. **c** The quantification of migration ability of U87 cells were presented as percentage of the wound area. The quantification of U87 (**d**) and U251 (**e**) cell invasion is presented as invasive cell numbers per field. qRT-PCR analysis of the mRNA expression levels of MMP1 (**f**), MMP2 (**g**), MMP9 (**h**) and MMP13 (**i**) in U87 and U251 cells with or without lncRNA PTCSC3 overexpression. Data are expressed as the means ± S.D of three independent experiments. “**” indicates *P* < 0.01

The expression level of lncRNA PTCSC3 in glioma was detected in human microglia cells, astrocyte and four glioma cell lines (U87, U251, SHG44 & SHG139) by qRT-PCR. LncRNA PTCSC3 was downregulated in glioma cell lines compared with human microglia cells and astrocyte (Fig. 1). Among the four glioma cell lines, U87 had the lowest expression level of lncRNA PTCSC3 compared with U251, SHG44 and SHG139 (Fig. 1). U87 and U251 were selected to investigate the biological function of lncRNA PTCSC3 in glioma cells. As lncRNA PTCSC3 was downregulated in glioma cell lines, the gain-of-function experiment was performed using lentivirus vector in the U87 and U251 glioma cell lines. The transfection efficiency was determined by qRT-PCR after 72 h of transfection. As shown in Fig. 2a, the expression levels of lncRNA PTCSC3 in U87 and U251 cells transfected with the lncRNA PTCSC3 overexpression vector were increased by 189.34-fold and 152.01-fold respectively, compared with control cells. In CCK8 assay, the cell viabilities of U87 and U251 cells were determined at five time points (0, 12, 24, 48, and 72 h) after being transfected with the lncRNA PTCSC3 overexpression vector. The viability of the U87 and U251 cells decreased significantly at 48, 72 h with overexpression of lncRNA PTCSC3 compared with the control (Fig. 2b, c). In cell cycle analysis, the proportions of cells in the G1 phase was significantly increased in U87 and U251 cells with lncRNA PTCSC3 overexpression compared with the control. Additionally, the proportion of cells in S and G2/M phases were reduced in the lncRNA PTCSC3 overexpression group (Fig. 3d-f). In colony formation assay, there were fewer colonies in the lncRNA PTCSC3 overexpression group compared with the control (Fig. 3g, i). Moreover, the apoptosis assay showed that lncRNA PTCSC3 overexpression promoted apoptosis in U87 and U251 cells (Fig. 4a–c). Our result indicated that lncRNA PTCS3 overexpression inhibited proliferation by inducing cell cycle arrest and apoptosis in glioma cells as well as suppressing cell viability.

### LncRNA PTCSC3 overexpression inhibits migration and invasion in glioma cells

Scratch test and transwell assay were used to evaluate the affect of lncRNA PTCSC3 on glioma cell migration and invasion respectively. The scratch test showed that lncRNA PTCSC3 overexpression remarkably inhibits U87 cell migration (Fig. 4a), which was quantified by the percentage of the wound area (Fig. 4c). The transwell invasion assay showed that overexpression of lncRNA PTCSC3 reduced the invasive capacity of U87 (Fig. 4b, d) and U251 cells (Fig. 4b, e). Further, the expression of matrix metalloproteinases (MMPs) in lncRNA PTCSC3 elevated cells were detected to investigate a potential mechanism for lncRNA PTCSC3 in the inhibition of migration and invasion, indicating that MMP2 and MMP9 expression were downregulated in lncRNA PTCSC3 overexpressed cells, while MMP1 and MMP13 remained unaffected (Fig. 4f–i). These data showed that lncRNA PTCS3 overexpression inhibited glioma cell migration and invasion via downregulating MMP2 and MMP9 expression.

### LncRNA PTCSC3 overexpression inhibits the epithelial-mesenchymal transition of U87 cells

**Fig. 5:**
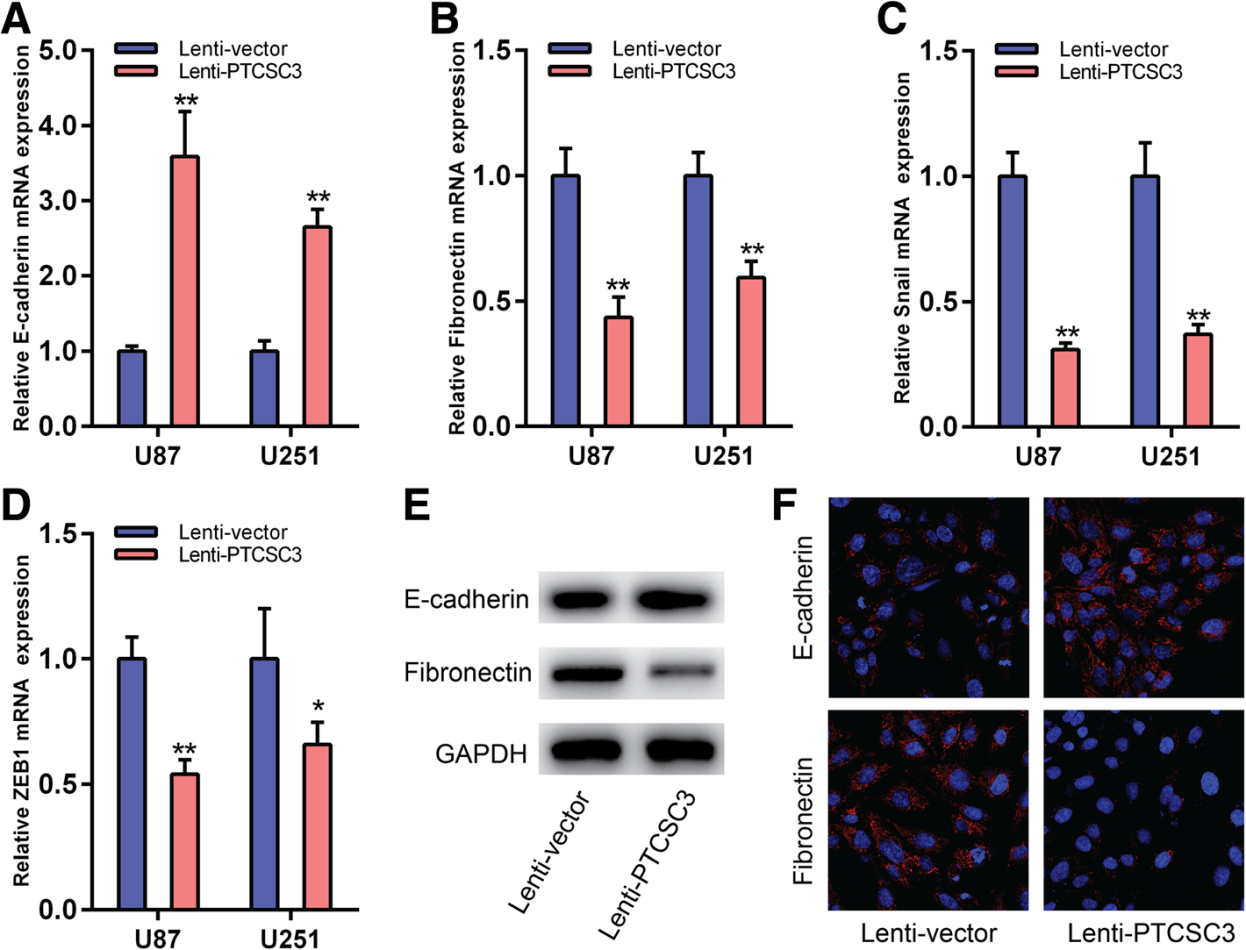
LncRNA PTCSC3 overexpression inhibits the epithelial-mesenchymal transition of U87 cells. qRT-PCR analysis of the mRNA expression levels of E-cadherin (**a**), Fibronectin (**b**), Snail (**c**) and ZEB1 (**d**) in U87 and U251 cells with or without lncRNA PTCSC3 overexpression. **e** Western blot analysis of E-cadherin and Fibronectin in U87 cells. GAPDH was used as a loading control. Representative images of three repeated experiments are presented. **f** Representative images of immunofluorenscence analysis of E-cadherin and Fibronectin in U87 cells. Original magnification 400 ×. Data are expressed as the means ± S.D of three independent experiments. “*” indicates *P* < 0.05, “**” indicates *P* < 0.01

To investigate the affect of lncRNA PTCSC3 on the epithelial-mesenchymal transition of glioma cells, the study examined the mesenchymal markers and epithelial markers in U87 cells with or without lncRNA PTCSC3 overexpression. It showed that E-cadherin (epithelial marker) was upregulated (Fig. 5a) while Fibronectin, Snail and ZEB1 (mesenchymal markers) were downregulated in lncRNA PTCSC3 overexpressed cells (Fig. 5b-d). The function of lncRNA PTCSC3 increased the expression of E-cadherin and reduced the expression of Fibronectin was further confirmed by western blot (Fig. 5e and Additional file 2: Figure S2) and immunofluorenscence (Fig. 5f).

### LncRNA PTCSC3 overexpression inhibits the Wnt/β-catenin signaling pathway by suppressing LRP6

**Fig. 6:**
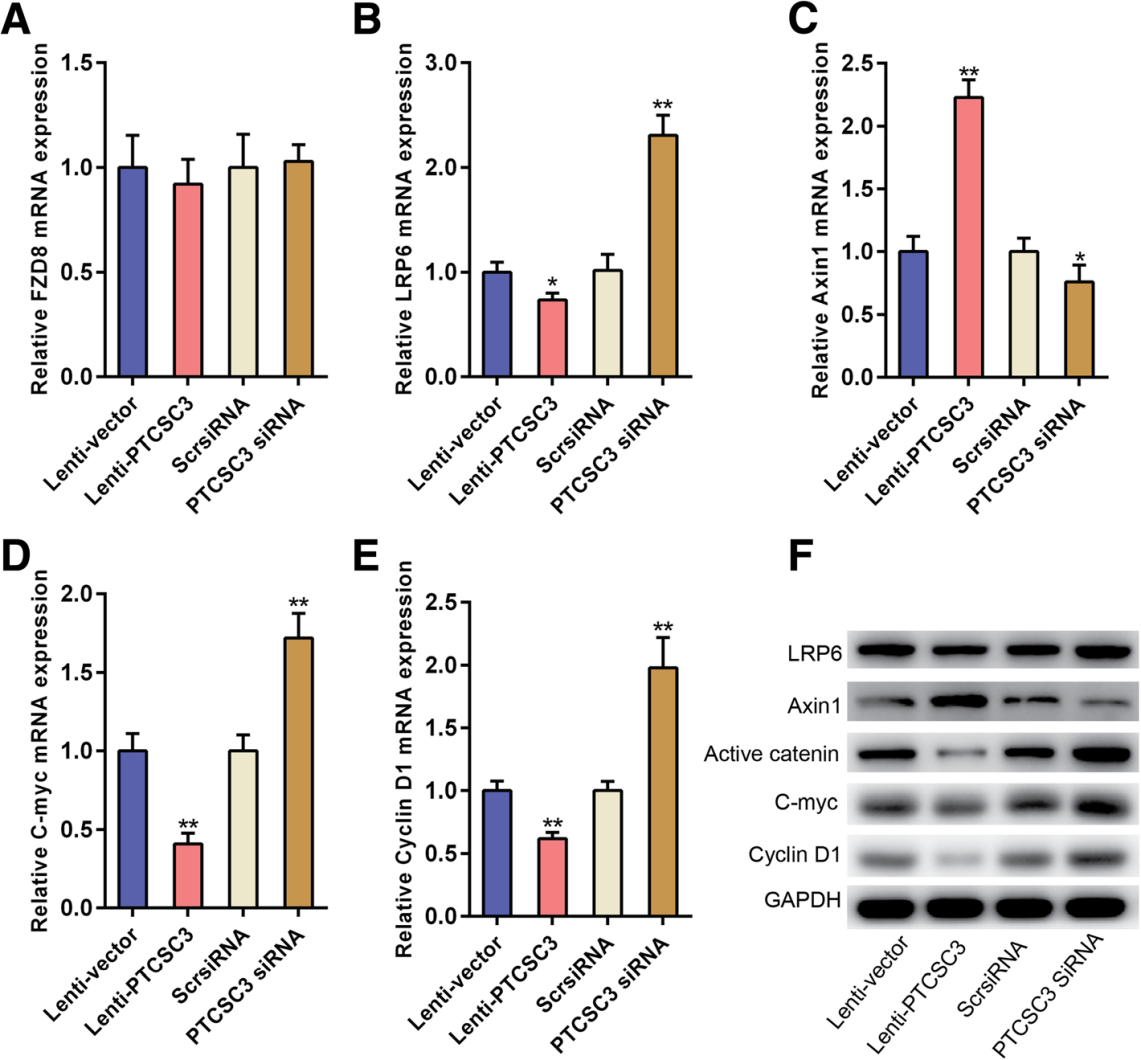
LncRNA PTCSC3 overexpression inhibits the Wnt/β-catenin signaling pathway by suppressing LRP6. qRT-PCR analysis of the mRNA expression levels of FZD8 (**a**), LRP6 (**b**), Axin1 (**c**), C-myc (**d**) and Cyclin D1 (**e**) in cells with overexpression or knockdown of lncRNA PTCSC3. **f** Western blot analysis of LRP6, Axin1, Active catenin, C-myc and Cyclin D1 in U87 cells. GAPDH was used as loading control. Representative images of three repeated experiments are presented. Data are expressed as the means ± S.D of three independent experiments. “*” indicates *P* < 0.05, “**” indicates *P* < 0.01

The affect of lncRNA PTCSC3 on the Wnt/β-catenin signaling pathway was investigated in U87 cell line. Data indicated that the expression of LRP6, C-myc and Cyclin D1 were downregulated and the expression of Axin1 was upregulated when lncRNA PTCSC3 was overexpressed, wherease the expression of FZD8 remained unchanged (Fig. 6a–e). Conversely, the expression of LRP6, C-myc and Cyclin D1 were induced while that of Axin1 was reduced when lncRNA PTCSC3 was knocked down, wherease FZD8 expression remained unchanged (Fig. 6a–e). In western blot analysis, lncRNA PTCSC3 overexpression downregulated LRP6 expression, subsequently suppressing the expression of active β-catenin, C-myc and cyclin D1, while increasing Axin1 expression, and vice versa (Fig. 6f and Additional file 3: Figure S3).

## Discussion

It is well-known that lncRNAs are dysregulated in a variety of tumors and play important roles in carcinogenesis as well as cancer progression [11]. In the present study, we detected the expression level of lncRNA PTCSC3 in glioma. We demonstrated that lncRNA PTCSC3 was downregulated in glioma cells compared with normal brain cells. To investigate the biological function of lncRNA PTCSC3, we performed gain-of-function experiments in glioma cell lines. These experiments indicate that overexpression of lncRNA PTCSC3 inhibited proliferation, migration and invasion in glioma cells. For the mechanism study, we explored the influences of dysregulated lncRNA PTCSC3 in the EMT and Wnt/β-catenin signaling pathways, revealing a negatively regulatory mechanism between these pathways.

Evidences are accumulating that lncRNAs play pivotal roles in various maligancies. LncRNA H19 was reported to be overexpressed in pancreatic ductal adenocarcinoma and plays oncogenic role through promoting cancer cell proliferation [12]. It was also recognized as a novel biomarker for diagnosis of gastric cancer in plasma [13]. Urothelial Carcinoma Associated 1 (UCA1), which acts as oncogenic lncRNA and promotes bladder cancer progression [14], has been studied in a meta-analysis that up-regulated UCA1 was significantly corrected with LNM and poor OS in patients with various cancers [15]. In glioma, the exploration of lncRNAs is still superficial. LncRNA-ATB was abnormally upregulated in glioma and played an oncogenic role in glioma cells by inhibiting miR-200a and facilitating TGF-β2 [16]. LncRNA HULC, which was highly upregulated in liver cancer, was discovered as an oncogene in glioma [17]. Another lncRNA MALAT1 (metastasis-associated lung adenocarcinoma transcript 1), which was initially found to be highly expressed in lung cancer and was a favorable prognostic factor for the survival of patients with stage I non-small-cell lung cancer (NSCLC), was demonstrated to have a tumor suppressive role in glioma by attenuating ERK/MAPK-mediated growth and MMP2-mediated invasiveness [18]. This is the first time that lncRNA PTCSC3 was reported in glioma. LncRNA PTCSC3 initially demonstrated that it was a susceptible gene in papillary thyroid carcinoma and that is also maintains the role of inhibition in thyroid cancer invasion [9, 10]. Our results showed that lncRNA PTCSC3 was downregulated in glioma cells. The analysis of tumor cell proliferation and invasion abilities in lncRNA PTCSC3-elevated cells showed the inhibitory role of lncRNA PTCSC3 in glioma, which is similar to its role in thyroid cancer.

To elucidate the possible mechanism by which lncRNA PTCSC3 regulates the proliferation and invasion of glioma cell, qRT-PCR and western blot analysis of the key molecular factors of EMT-associated biomarkers, including E-cadherin, fibronectin, Snail and ZEB1, were assessed in our study. EMT is a process involved in embryonic development, during which cells lose their epithelial features and adopt characteristics of mesenchymal cells. The EMT also plays a role in cancer invasion and metastasis in a sequence of discrete steps [19, 20]. Decreased expression of the epithelial marker E-cadherin and increased expression of mesenchymal markers, such as N-cadherin and fibronectin, are the most important features of EMT [21]. Transcriptional repressors Snail and ZEB have critical roles in regulating the EMT [22–24]. The activation of these transcriptional factors results in induction of the EMT phenotype [25]. Among the EMT markers we evaluated, the overexpression of lncRNA PTCSC3 led to the downregulation of E-cadherin and upregulation of Fibronectin, Snail and ZEB1, indicating that overexpression of lncRNA PTCSC3 represses the EMT.

EMT activation is induced by a variety of signaling pathways [26–28]. Here, we investigated the Wnt/β-catenin signaling pathway in glioma cell lines. Wnt signals are transduced across the membrane by low-density lipoprotein receptor–related protein (LRP) receptors [29]. According to our result, LRP6 was inactivated when lncRNA PTCSC3 was overexpressed. The APC/Axin/GSK/β-catenin-complex has been called a “destruction complex” in Wnt signaling [30]. In normal conditions, the complex remains stable. When Axin dissociates, β-catenin translocates to the nucleus and activates the transcription of specific target genes, such as c-Myc and cyclin D1 [31]. Previous reports indicate that the separation of the “destruction complex” leads to an increase in Axin1 protein expression and a concomitant decrease in β-catenin the cellular environment [32]. Our data revealed results similar to previous studies indicating that dissociation of the complex resulted in an increase in Axin1 and a reduction in active β-catenin as well as the subsequent inactivation of c-Myc and cyclin D1. The dissociation of the “destruction complex” was initiated by the inactivation of LRP6, which was primarily controlled by the overexpression of lncRNA PTCSC3.

Matrix metalloproteinases (MMPs) digest extracellular substrates and promote invasion of blood vessels and lymphatics in the spread of cancers [33]. MMP2 and MMP9 are induced by the EMT pathway and promote post-EMT invasion of underlying tissues [21]. Another study also generalized that MMP-2 and MMP-9 are activated by Snail [34]. Moreover, MMPs degrade E-cadherin, disrupting adhesive junctions, to facilitate the EMT [35]. Unsurprisingly, this study showed that MMP2 and MMP9 expression was downregulated in lncRNA PTCSC3-overexpressing cells, concomitant with the elevated expression of E-cadherin.

Our study illuminated a complicated mechanism network of how lncRNA PTCSC3 regulated glioma progression. As it was indicated, Wnt/β-catenin signaling pathway was initiated by LRP6, which was the target of lncRNA PTCSC3; the “destruction complex” was separated, resulting in a series of downstream alterations (Axin1, C-myc, Cyclin D1, etc.). The activation of Wnt/β-catenin signaling pathway induced EMT activation, which could also be regulated by Snail and ZEB1. The expression of MMP2 and MMP9 were downregulated, which was induced by the activation of EMT. In return, the decreased expression of MMP2 and MMP9 could also lead to an accumulation of E-cadherin.

## Conclusion

In conclusion, lncRNA PTCSC3 is downregulated in glioma cells. The overexpression of lncRNA PTCSC3 inhibits proliferation, migration and invasion of glioma cells and suppresses the Wnt/β-catenin signaling pathway through targeting LRP6. LncRNA PTCSC3 is a potential novel therapeutic target for intervention of glioma.

## Additional files

Additional file 1: Figure S1. qRT-PCR analysis of lncRNA PTCSC3 expression levels in U87 transfected with ScrsiRNA or PTCSC3 siRNA. Data are expressed as the means ± S.D of three independent experiments. “**” indicates *P* < 0.01. (TIF 40 kb)
Additional file 2: Figure S2. The protein expression level of E-cadherin and Fibronectin in U87 cells with or without lncRNA PTCSC3 overexpression was quantified. Data are expressed as the means ± S.D of three independent experiments. “**” indicates *P* < 0.01. (TIF 47 kb)
Additional file 3: Figure S3. The protein expression levels of LRP6 (A), Axin1 (B), Active catenin (C), C-myc (D) and Cyclin D1 in cells with overexpression or knockdown of lncRNA PTCSC3. Data are expressed as the means ± S.D of three independent experiments. “**” indicates *P* < 0.01. (TIF 749 kb)

## Abbreviations

CCK-8: Cell counting kit-8
DMEM: Dulbecco’s Modified Eagle Medium
EMT: Epithelial-mesenchymal transition
FBS: Fetal bovine serum
FZDl: Frizzled class receptor 8
GAPDH: Glyceraldehyde-3-phosphate dehydrogenase
LncRNA PTCSC3: Long noncoding RNA papillary thyroid carcinoma susceptibility candidate 3
LRP6: Low-density lipoprotein receptor–related protein 6
MMP: Matrix metalloproteinase
qRT-PCR: Quantitative reverse transcriptase-polymerase chain reaction
ZEB1: Zinc finger E-box binding homeobox 1
