# Supplementary figures and images for: Long noncoding RNA papillary thyroid carcinoma susceptibility candidate 3 (PTCSC3) inhibits proliferation and invasion of glioma cells by suppressing the Wnt/β-catenin signaling pathway
Source: BMC Neurol. 2017 Feb 10;17:30. doi: 10.1186/s12883-017-0813-6 (PMC5303216; doi:10.1186/s12883-017-0813-6)

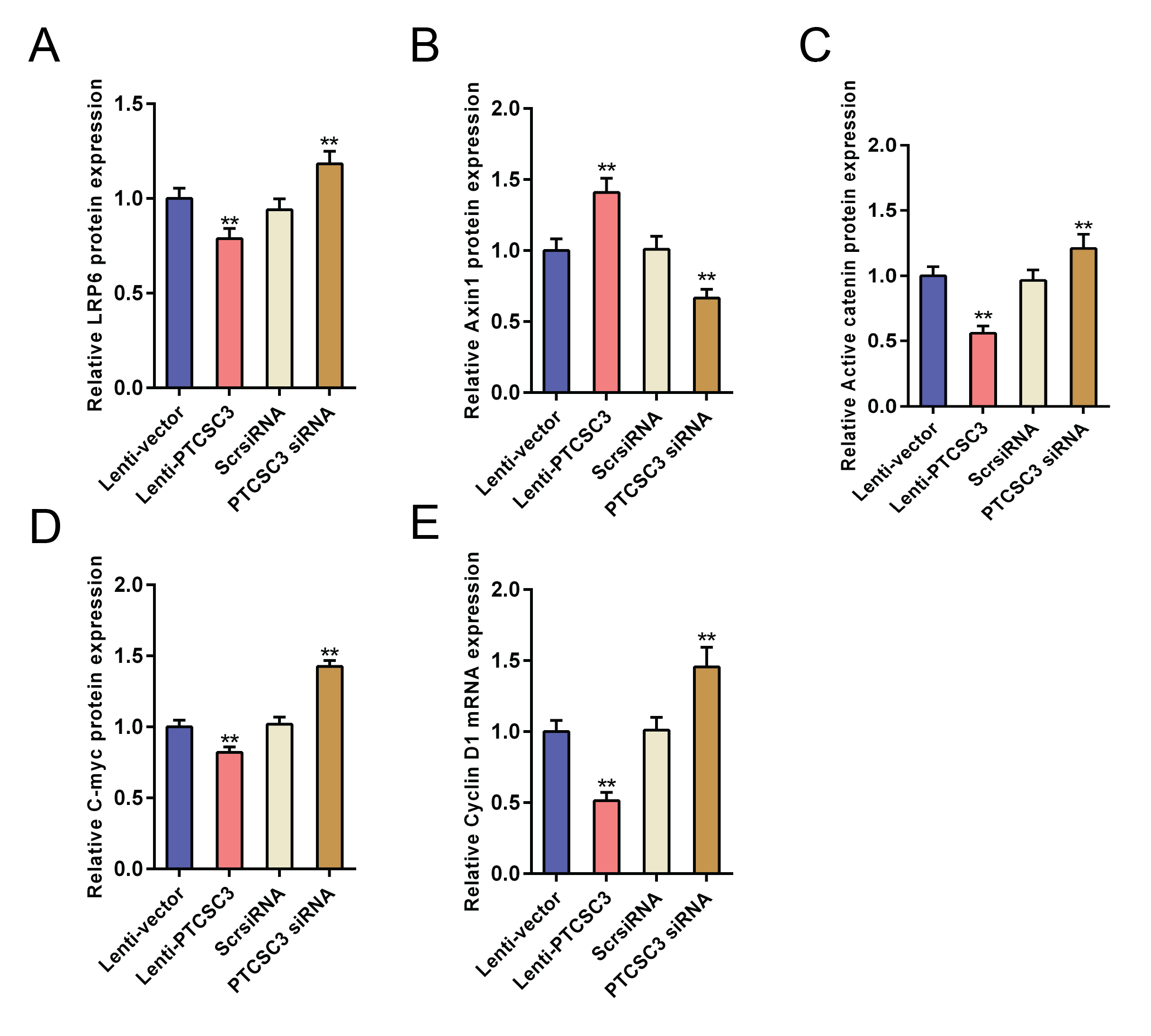

Supplement: Additional file 3: Figure S3. — The protein expression levels of LRP6 (A), Axin1 (B), Active catenin (C), C-myc (D) and Cyclin D1 in cells with overexpression or knockdown of lncRNA PTCSC3. Data are expressed as the means ± S.D of three independent experiments. “**” indicates P < 0.01. (TIF 749 kb) [file 12883_2017_813_MOESM3_ESM.tif]
